# SEMbap: Bow-free covariance search and data de-correlation

**DOI:** 10.1371/journal.pcbi.1012448

**Published:** 2024-09-11

**Authors:** Mario Grassi, Barbara Tarantino

**Affiliations:** Department of Brain and Behavioral Sciences, University of Pavia, Pavia, Italy; University of Southern California, UNITED STATES OF AMERICA

## Abstract

Large-scale studies of gene expression are commonly influenced by biological and technical sources of expression variation, including batch effects, sample characteristics, and environmental impacts. Learning the causal relationships between observable variables may be challenging in the presence of unobserved confounders. Furthermore, many high-dimensional regression techniques may perform worse. In fact, controlling for unobserved confounding variables is essential, and many deconfounding methods have been suggested for application in a variety of situations. The main contribution of this article is the development of a two-stage deconfounding procedure based on Bow-free Acyclic Paths (BAP) search developed into the framework of Structural Equation Models (SEM), called SEMbap(). In the first stage, an exhaustive search of missing edges with significant covariance is performed via Shipley d-separation tests; then, in the second stage, a Constrained Gaussian Graphical Model (CGGM) is fitted or a low dimensional representation of bow-free edges structure is obtained via Graph Laplacian Principal Component Analysis (gLPCA). We compare four popular deconfounding methods to BAP search approach with applications on simulated and observed expression data. In the former, different structures of the hidden covariance matrix have been replicated. Compared to existing methods, BAP search algorithm is able to correctly identify hidden confounding whilst controlling false positive rate and achieving good fitting and perturbation metrics.

## Author summary

Directed acyclic graphs (DAGs) directed graph, with variables at the vertices and direct causal connections at the edges, can be used to illustrate the causal structure of the SEM, but this does not always mean that all significant factors are considered. We examine a class of models that may include some hidden variables. Specifically, we consider that the graph represents a bow-free acyclic path diagram (BAP), where the directed edges signify direct causal effects, while the bidirected edges suggest hidden confounders. In this paper, we provide a two-step deconfounding technique based on BAP search, which is included into the SEM framework via the SEMbap() function implemented in the R package **SEMgraph**. Secondly, we want to offer a significant evaluation of the most advanced deconfounding techniques using both synthetic and real data, as well as knowledge of a biological signaling pathway encoded in a DAG, in terms of (i) SEM fitting, (ii) system perturbation, and (iii) recovery performance metrics. The BAP search algorithm outperforms current techniques in accurately detecting hidden confounding, regulating false positive rate, and producing well-fitting and perturbation metrics.

## Introduction

Large-scale gene expression and genotype data are now being produced at an unprecedented rate thanks to rapidly developing experimental methods for testing genomic data. Studies of gene expression at a large scale are frequently affected by biological and technical sources of expression variation, such as batch effects, sample characteristics, and environmental influences. The ability of researchers to quantify interesting biological signals can be enhanced by recognizing and removing these potential confounders. Confounding factors might be established sources of expression variance (known covariates) or developed empirically from the expression dataset (hidden covariates).

Unobserved confounders might make it difficult to learn the causal connections between observable variables, particularly when those confounders are “pervasive” (i.e., they affect a large number of observed variables).

Gene co-expression and confounding factors both generate patterns of gene correlation, making it difficult to discern between the two and leading to possible erroneous correlations between a lot of different variables [1, 2].

In addition, the presence of unobserved confounding factors that impact both the predictors and the outcome, the performance of many high-dimensional regression approaches may deteriorate. Directed Acyclic graphs (DAGs) encoded in linear Structural Equation Models (SEM) assume casual sufficiency [3] that requires no hidden (or latent) variables that are common causes of two or more observed variables; i.e., the covariance matrix of the unobserved terms is diagonal. This assumption is particularly constraining, and unrealistic in most applications.

In fact, adjusting for unobserved confounding variables is crucial, and different deconfounding techniques have been proposed for use in diverse contexts [2, 4, 5].

Standard high-dimensional regression methods assume that the underlying coefficient vector is sparse (i.e., the response is only affected by a few predictors) [6, 7]. However, when there is confounding in a linear model, in addition to the few observed predictors that can influence the response, there are more hidden predictors that are associated with the outcome. Some methods for relaxing the sparsity assumption represent the structure of the regression parameter as the sum of a sparse and a dense vector. The real underlying regression vector will be altered by some modest, dense perturbation if the confounding factors have an impact on a large number of predictors [8].

The approaches frequently employ some form of Principal Component Analysis (PCA) defined by Singular Value Decomposition (SVD) to estimate the confounding variables directly from the data. If dense latent factors exist, the initial main components are distinct from the others, and a two-step procedure is performed computing normalized residuals prior to downstream regression analysis. [9] and [10] suggest multiplying response vector and the predictor matrix leftward by a well selected spectrum transformation matrix, which modifies the singular values of input data. [9] proposes the Lava estimator whilst [10] suggests straightforward spectral transformation known as the “trim” transform, which is perhaps slightly easier to use than the Lava or the PCA adjustment estimator but less smoother than the former. After that, the altered data matrix may be utilized as the input for a high-dimensional sparse regression method, of which the LASSO is a prime example.

In graphical models [11, 12], the goal is to estimate a concentration matrix, i.e. the inverse of the covariance matrix, of the observed variables, that in the confounding scenario is often not sparse. [13] addresses the issue of calculating the precision matrix in the presence of a few hidden confounding factors by decomposing the concentration matrix into a sparse matrix and a low-rank matrix for revealing the conditional graphical model structure in the observable variables as well as the number and impact of the hidden variables. Low Rank plus Sparse (LRpS) decomposition algorithm removes unwanted variation by using the Alternating Direction Method of Multipliers (ADMM) algorithm [14]. Unlike the other methods, the decomposition in [13] regards the entire precision matrix and not only the regression coefficient. In causal structure learning, a two-step approach is suggested by [15] which first remove the effect of the hidden variables by LRpS and then estimates the Completed Partially DAG (CPDAG) under the assumption of causal sufficiency by using the estimated sparse covariance matrix of LRpS.

[16] introduces a novel computational approach in DAG gene expression application, known as Differential Causal Effects (DCEs), which contrasts healthy cells with cancerous cells using Average Causal Effects (ACEs); i.e. the total effect of a source-sink link of a SEM. The technique enables for the detection of specific edges in a signaling pathway that are dysregulated in cancer cells while controlling for confounding. The authors extend the linear function representing the *sink* ∼ *source*+ *parent*(*source*) equation by including the first q principal components of the design matrix as additional source variables.

The main contribution of this article is the development of a two-stage deconfounding procedure based on Bow-free Acyclic Paths (BAP) search developed into the framework of SEM called SEMbap() and implemented in the R package SEMgraph [17]. A BAP is a acyclic graph that can have directed and bidirected edges, where the directed edges represent direct causal effects encoded by regression coefficients, and the bidirected edges represent hidden confounders encoded by pairwise covariances. The bow-freeness condition means there cannot be both a directed and a bidirected edge on the same pair of variables. Our approach assumes arbitrary latent confounding, i.e. latent variables (LVs) induce confounding dependencies among the observed variables with bow-free covariances if arbitrarily exists at least one pair or many pairs of variables with covariances not equal zeros. This assumption is substantially weaker than the latent denseness, where few hidden variables have direct effect on many of the observed variables, as required from the previous cited methods. As a result, there’s the need to find an optimal solution for both arbitrary and pervasive confounding scenarios.

A second objective is to provide a meaningful comparison of the state-of-the-art deconfounding methods on real and synthetic data and on a *priori* knowledge of a biological signalling pathway encoded in a DAG in terms of (i) SEM fitting, (ii) system perturbation and (iii) recovery performance metrics. Simulating different confounding scenarios, we want to understand if our methodology could be an optimal solution for different scenarios.

The rest of the article is divided into the following sections. First, both the inference process and the user interface for SEMbap() features with respect to gene expression data are described. The experimental setup for assessing deconfounding techniques is then described, including the simulation design and real data application. Finally, we present the findings and a concluding analysis.

## Materials and methods

### SEM

A linear SEM specifies a causal mechanism underlying a set of variables [18, 19]. Each variable is defined as a linear combination of a subset of the remaining variables, with the addition of an error term. The linear equations involving the variables, *Y*_*i*_ = (*Y*_*i*1_, …, *Y*_*ip*_)^*T*^ and unobserved terms *U*_*i*_ = (*U*_*i*1_, …, *U*_*ip*_)^*T*^ can be expressed in matrix form as follows:

$$\begin{array}{c} Y_{i}=BY_{i}+U_{i},\;\;\;\text{with}\;\;\;\text{cov}\left(U_{i}\right)=\Psi \end{array}$$ (1)

where *B*(*p*, *p*) is a real matrix, Ψ(*p*, *p*) is a positive semi-definite matrix. Usually is assumed that all variables, *Y*_*i*_ have been standardized to mean zero and variance one, and are independent and identically distributed (i.i.d.) across the indices *i* = 1, …, *n*.

A SEM has associated a mixed graph, *G* = (*V*, *E*), where *V* is the set of nodes (i.e., variables) and *E* is the set of edges (i.e., connections) that reflects the structure of *B* and Ψ. For every non-zero entry *B*_*jk*_ there is a directed edge from variable *k* to variable *j* (*k* → *j*), and for every non-zero entry Ψ_*jk*_ there is a bidirected edge between variable *j* and variable *k* (*j* ↔ *k*). A directed edge indicates that *k* is an explanatory variable for response variable *j*. A bi-directed edge indicates that errors between variable *j* and variable *k* are dependent, which is assumed when there exists an unobserved (i.e., latent) confounder between *j* and *k*.

The mixed graph, also known as a *path diagram* [20, 21], is a formal tool to evaluate the hierarchical structure of a system, where we can identify *exogenous variables*, having zero explanatory variables in all structural equations, and *endogenous variables*, having at least one explanatory variable in at least one structural equation.

In graph theory, exogenous variables are *source* nodes, with incoming connectivity equal to 0, whilst endogenous variables are nodes with non-zero incoming connectivity. Endogenous variables can be further divided into *connectors*, with non-zero outgoing connectivity, and *sinks*, having no outgoing connections.

We consider three special types of SEM:

- Directed Acyclic Graphs (DAGs) used in causal inference [22], where loops are not allowed; i.e., *B* defines a lower (or upper) triangular weighted adjacency matrix, and all covariances are null: *ψ*_*jk*_ = 0 and Ψ = *diag*(*ψ*_1_, …, *ψ*_*p*_).
- Bow-free Acyclic Paths (BAPs), *B* has an acyclic structure, and bidirected connections (covariances) in Ψ are not null only if do not share any directed link: if *β*_*jk*_ = 0 then *ψ*_*jk*_ ≠ 0; i.e., they are bow-free [23].
- Latent (or Hidden) Variable Graphs (LVs), *B* has an acyclic structure, and *U* terms encode a Factor Analysis (FA) model [24] with common latent factors and specific errors: *U*_*i*_ = Γ*F*_*i*_+ *E*_*i*_, where Γ(*p*, *q*) are the loading factors of *q* < *p* LVs (i.e., new exogenous or source variables), *F*_*i*_ = (*F*_*i*1_, …, *F*_*iq*_)^*T*^ and *E*_*i*_ = (*E*_*i*1_, …, *E*_*ip*_)^*T*^ are idiosyncratic error terms.

The multivariate system (1) is equivalent to *Y*_*i*_ = (*I*−*B*)^−1^*U*_*i*_ that links observed variables, *Y*_*i*_ only on unobserved variables, *U*_*i*_ with the population covariance matrix of the observed variables, $\Sigma ≔E(Y_{i}Y_{i}^{T})$ for DAG, BAP or LV models given by:

$$\begin{array}{c} \Sigma_{1}={\left(I-B\right)}^{-1}D_{\psi }{\left(I-B\right)}^{-T} \end{array}$$ (2)

$$\begin{array}{c} \Sigma_{2}={\left(I-B\right)}^{-1}\Psi {\left(I-B\right)}^{-T} \end{array}$$ (3)

$$\begin{array}{c} \Sigma_{3}={\left(I-B\right)}^{-1}\left(\Gamma \Gamma^{T}+D_{e}\right){\left(I-B\right)}^{-T} \end{array}$$ (4)

Considering *U*_*jk*_ an unobserved confounder between pair of observed variables, the covariance matrices Ψ_1_ = *D*_*ψ*_, Ψ_2_ = Ψ and Ψ_3_ = ΓΓ^*T*^ + *D*_*e*_ account for unobserved confounding, that we call *de-correlated, arbitrary* or *pervasive* confounding, respectively.

By definition, DAG is a de-correlated model, BAP model states that the LVs induce confounding dependencies between at least one pair of observed variables (*Y*_*j*_, *Y*_*k*_), and LV model assumes that several unobserved variables have an effect on many of the observed ones. To note, not every *Y*_*i*_ needs to be affected by each LV.

Generally, in the SEM framework, free (i.e., unknown) parameters, (*B*, Ψ) are computed by Maximum Likelihood Estimation (MLE), assuming all model variables as jointly Gaussian, so that the implied covariance matrix, Σ is close to the observed sample covariance matrix, *S* ([18], p. 135). This is obtained by maximizing the model log-likelihood function, log*L*(*B*, Ψ) given data, that is equivalent to the Weighted Least Square (WLS) procedure, if the SEM is a DAG, i.e. with a de-correlate model (see Eq (10).

Therefore, we propose a novel two-stage de-correlation approach based on BAP search as the initial step, and BAP deconfounding as the subsequent step. The first step employs the following: (i) the d-separation tests are conducted between all pairs of variables with missing connections in the DAG using the Shipley’s basis sets, as outlined in [25]. Alternatively, (ii) the conditional independence (CI) tests are applied with a glasso search, as detailed in [26], when the DAG is of considerable size. In the second step, the following is employed: (i) the precision matrix, fitted by Constrained Gaussian Graphical Model (CGGM) ([27], pg. 631), with the null (zero) pattern corresponding to the DAG edges and null (zero) bow-free covariances, is removed from the input data. Alternatively, (ii) the first principal component scores of the Graph Laplacian PCA (gLPCA) [28] connected to the bow-free covariances are added to the input data. In the next sections, we will provide a more detailed account of the BAP search, and BAP deconfounding (CGGM-based and gLPCA-based) procedures.

### BAP search

DAGs are increasingly used in many areas of sciences and engineering for visual representations of causal hypotheses [3, 29]. By making underlying relations explicit, they can enable us to determine whether confounding is present for the current causal question. In the causal DAG method, a connection between two variables denotes causation; variables without a clear causal relationship are left unconnected. Missing edges in causal network inference using a DAG are frequently hidden by unmeasured confounding variables. It is possible to think of a latent variable (LV) acting on both variables when there is a missing edge between them.

In a DAG, missing edges between nodes imply a series of independence relationships between variables (either direct or indirect). These independences are implied by the topology of the DAG and are determined through d-separation: two nodes, *Y*_*j*_ and *Y*_*k*_, are d-separated by a set of nodes *S* if conditioning on all members in *S* blocks all confounding (or *backdoor*) paths between *Y*_*j*_ and *Y*_*k*_ [30, 31].

We need to define: (i) a path that begins with an arrow pointing to *Y*_*k*_ and ends with an arrow pointing to *Y*_*j*_, called a confounding (or back-door) path from *Y*_*k*_ to *Y*_*j*_ (*Y*_*k*_ ← … → *Y*_*s*_); (ii) a node *Y*_*s*_ ∈ *S* in which two arrowheads meet *Y*_*s*_ (→*Y*_*s*_←) called a collider; (iii) a collider along a path blocks (close) that path. However, conditioning on a collider (or any of its descendants) unblocks (open) that path; (iv) blocking a confounding path requires conditioning on any intercepted (not-collider) nodes on the path.

With these definitions out of the way, two nodes *Y*_*k*_ and *Y*_*j*_ are d-separated by *S* if conditioning on all members in *S* blocks all confounding paths between the two nodes. As outlined in [25] if *Y*_*j*_ has a higher causal order than *Y*_*k*_, it is possible to find a minimal set, *S* implying all the other possible independences defined by a basis set: *S*_*U*_ = {*Y*_*j*_ ⊥ *Y*_*k*_|pa(*j*) ∪ pa(*k*), *j* > *k*}, where pa() is the “parent” set; i.e., the variables with a direct effect on the response variable in a DAG. The number of d-separation constraints in the set *S*_*U*_ equals the number of missing edges, corresponding to the number of degrees of freedom (df) of the model. If the graph is not very large with huge missing edges, it is possible to perform local testing of all missing edges separately, using the Fisher’s z-transform of the partial correlation. An edge (*j*; *k*) is absent in the graph when the null hypothesis:

$$\begin{array}{c} H_{0}:\;\rho_{jk.U}=\text{cor}\left(Y_{j};Y_{k}\;|\;\text{pa}\left(j\right)\cup \text{pa}\left(k\right)\right)=0 \end{array}$$ (5)

is not rejected. Because the individual tests implied by the basis set, *S*_*U*_ are mutually independent, each one can be tested separately at a significance level of *α*, after multiple testing correction following a Bonferroni or False Discovery Rate (FDR) procedure. In this way, lack-of-fit in the whole model can be decomposed into lack-of-fit involving pairs of variables.

If the number of missing edges is large, only those tests where the number of conditioning variables does not exceed a given value can be performed. In high-dimensional conditional independence tests can be very unreliable, and we suggest to force the sparsity by testing bow-free covariances with basis set size close to the sparsity index, $s=\sqrt{n}/log\left(p\right)$ [32].

Alternatively, with a huge input DAG, Gaussian Graphical Model (GGM) can be applied [33]. In GGM statistical inference is based on conditional dependence of pairwise variables (*Y*_*j*_;*Y*_*k*_) given the conditional set *rest* = *Y*_−(*j*;*k*)_ defined by all variables in the graph excluding (*Y*_*j*_;*Y*_*k*_):

$$\begin{array}{c} H_{0}:\;\rho_{jk.R}=\text{cor}\left(Y_{j};Y_{k}\;|rest\right)=0 \end{array}$$ (6)

The pairwise partial correlations, *ρ*_*jk*.*R*_ are reflected in the elements of the precision matrix; i.e., the inverse of the covariance matrix, *Ω* = Σ^−1^. Specifically:

$$\begin{array}{c} \rho_{jk.R}=\frac{-\omega_{jk}}{\sqrt{\omega_{jj}\omega_{kk}}}=0\;\;\Leftrightarrow \;\;\omega_{jk}=0 \end{array}$$ (7)

Thus the sparsity pattern of *Ω* contains the pairwise Conditional Independence (CI) relations encoded in the corresponding precision graph, and the problem of estimating a GGM is equivalent to the problem of estimating *Ω*.

We point out that, testing for *H*_0_ in (6) is not necessarily equivalent testing for *H*_0_ in (5), because the conditioning set (6) can include both the common ancestors and descendants of (*Y*_*j*_;*Y*_*k*_). We set the DAG edges to zero, and assume that the conditioning set in (6) includes the common ancestors of nodes (*Y*_*j*_;*Y*_*k*_), with conditional effects of common descendants close to zero.

Under these conditions, for high-dimensional data (*n* ≪ *p*) the CI evaluation can be performed with a constrained graphical LASSO (glasso) procedure [26], a sparse penalised maximum likelihood estimate (pMLE) of the precision matrix $\hat{\Omega }$. The LASSO penalty, defined by a tuning parameter, *λ* applied to all elements of *Ω* promotes sparsity and can yield shrinkage estimates equal to ${\hat{\omega }}_{jk}=0$, indicating that only a few of them are non-zero bow-free covariances.

In summary, BAP search utilises d-separation or CI tests by adding a bidirected edge (i.e., bow-free covariance) to the DAG. The selected bidirected edges, which are encoded in the covariance matrix, Ψ provide information about which part of a DAG is not supported by the observed data. Although bidirected edges do not indicate a specific direction of causality, they identify the local misspecification resulting from the structural assumptions implied by the DAG, which may substantially alter the observed data variability. Consequently, selected bow-free covariances must be removed prior to the analysis (fitting) of a causal DAG.

### CGGM deconfounding

Bow-free covariances represent, for example, biomarkers that are not included in experimental chips, environmental variables, and underlying populations among experimental samples. It is unfortunate that such shared masking factors are often not directly measured in experiments despite their potential influence on measurements. Assuming that the BAP represents a good compromise between map accuracy and unidentified factors, and the implied population precision matrix Ψ^−1^ is known. Consequently, the observed variables, *Y*_*i*_, in the multivariate system (1) can be adjusted (or de-correlated) by means of Mahalanobis’s transformation (or Mahalanobis’s whitening) [34]:

$$\begin{array}{c} Z_{i}=\Psi^{-1/2}Y_{i}=\left(VL^{\frac{1}{2}}V^{T}\right)Y_{i} \end{array}$$ (8)

where Ψ^−1^ = *VLV*^*T*^ is the spectral decomposition of the precision matrix, with *V*(*p*, *p*) the matrix of the eigenvectors of Ψ^−1^, and *L*(*p*, *p*) the diagonal matrix of the corresponding eigenvalues. The new SEM is now:

$$\begin{array}{c} \Psi^{-1/2}Y_{i}=\Psi^{-1/2}\left(B\;Y_{i}+U_{i}\right)=A\;Z_{i}+D_{i} \end{array}$$ (9)

where *A* = Ψ^−1/2^*BΨ*^1/2^, *D*_*i*_ = Ψ^−1/2^*U*_*i*_, and cov(*D*_*i*_) = *I*_*p*_.

The Mahalanobis’s transformation reduces a BAP to a DAG. It follows that the log-likelihood of the model with a multivariate Gaussian distribution the Mahalanobis norm, i.e. the weighted squared L2-loss, is equivalent to the SEM log-likelihood [35]:

$$\begin{array}{c} \text{log}L\left(B,\Psi \right)\equiv -E(||\Psi^{-1/2}{\left(Y-BY\right)||}_{2}^{2}{)=-||Z-AZ||}_{2}^{2} \end{array}$$ (10)

Removing bow-free covariances helps to better train a DAG model, which assumes independence among error terms, and to perform Maximum Likelihood Estimate (MLE) of regression coefficients with equation-by-equation (nodewise) Ordinary Least-Squares.

When the population precision matrix is unknown, the adjusted (de-correlate) variables, *Z*_*i*_ should be computed from data by BAP (or bow-free covariance) search, as outlined in the previous Section BAP search.

The precision matrix, *Ω* = Ψ^−1^ can be fixed by setting the null (zero) patterns corresponding to the DAG edges and null (zero) edges after local d-separation screening in Eq (5). This allows parameters of the precision matrix to be estimated using a Constrained Gaussian Graphical Model (CGGM), which is a solution of the constrained log-likelihood maximisation problem.:

$$\begin{array}{c} \text{log}L\left(\theta \right)=\text{log}\;\text{det}\;\Omega {\left(\theta \right)}^{-1}-\text{tr}\left[\Omega \left(\theta \right)S\right]-\sum_{(j,k)\notin E}\gamma_{jk}\Omega {\left(\theta \right)}_{jk} \end{array}$$ (11)

where *S* is the observed covariance matrix, the set of free parameters are the non-zero structure defined by the know edges, *θ* = *E* and the Lagrange constants, *γ*′*s* constrain all missing edges. The minimization of the objective function is implemented in fitConGraph() function of the **ggm** R package [36], with the procedure originally described in “The Elements of Statistical Learning” ([27], pg. 631).

Alternatively, for huge DAGs we suggest to perform the two-step (BAP+CGGM) procedure via the algorithm in glasso() function of the **glasso** R package [37], fixing the tuning parameter, $\rho =\sqrt{log(p)/n}$. glasso() function also includes the option to estimate a constrained graph with missing edges by specifying which edges are fixed zeroes for some elements, while regularization on the other elements is activated. Given that the confounding variables in a arbitrary regime is encoded in missing edges of a *priori* DAG, we built the glasso graph if ${\hat{\omega }}_{jk}\neq 0$, fixing to zero the DAG structure. Thus, the DAG edges are guaranteed to be absent in the resulting constrained graph, and the edge set of bow-free covariances is defined by the set of all pairs (*Y*_*j*_;*Y*_*k*_) with nonzero elements in the estimated precision matrix.

Successively, the adjusted (de-correlated) data, removing the latent triggers responsible for the nuisance edges, are obtained by Mahalanobis’s transformation of Eq (8) with the square root of the precision matrix estimated by constrained “ggm” or “glasso”, $Z_{i}={\hat{\Omega }}^{1/2}Y_{i}$. Using the de-correlated data as additional information might enhance the DAG fitting that is represented in matrix *B*. Since the confounding correlation in *Z* vanishes, we find that this de-correlation step (see Section Experimental design) is able to substantially increase DAG goodness-of-fit indices, applying the best trade-off between global model fitting and local statistical significance of regression coefficients.

### gLPCA deconfounding

Latent (or Hidden) Variable Graph in SEM population covariance matrix of Eq (4) encodes a pervasive confounding. Component analysis is a common technique used in deconfounding methods to directly estimate pervasive confounding variables from the data, which appear in a number of economic and biological applications [38–40]. In a dense confounding regime the initial principal components are different from the others, and measuring confounding proxies for hidden variables as the scores of the first *q* principal components, *P* is a possible procedure [16]. This defines a SEM:

$$\begin{array}{c} Y_{i}=BY_{i}+\Gamma P_{i}+U_{i},\;\;\text{with}\;\;\text{cov}\left(U_{i}\right)=D_{\psi };\;\;\text{cov}\left(P_{i}\right)=I_{q} \end{array}$$ (12)

Of course, we have that cov(*Y*) = Σ_3_ of equation (4). The principal components are additional uncorrelated source nodes in the DAG, *G* = (*V* = (*V*_*p*_;*V*_*y*_), *E* = (*E*_*p*_;*E*_*y*_)), and the adjusted data matrix is the augmented matrix, *Z* = cbind(*P*, *Y*).

Computational aspect uses routine software. Standard PCA learns the projections or principal components of a dataset, *Y*(*n*, *p*) on *q*-dimensional orthonormal basis, *Q*(*p*, *q*) where *q* < *p*. PCA issue, though non-convex, has a global minimum that can be calculated using Singular Value Decomposition (SVD). Following [10], let *Y* = *PDQ*^*T*^ be the SVD of *Y*(*n*, *p*), where *P*(*n*, *r*), *Q*(*p*, *r*), *D*(*r*, *r*) = *diag*(*d*_1_ ≥ *d*_2_ ≥ … ≥ *d*_*r*_) are the spectral matrices with *P*^*T*^*P* = *Q*^*T*^*Q* = *I*_*r*_, and *r* = *min*(*n*;*p*) is the rank of *Y*. Then, the low-rank representation of the data, $\hat{Y}=PQ^{T}$ where *P*(*n*, *q*) = *YQ* is the projected data; i.e., the principal component scores.

PCA deconfounding assumes that confounding is dense, but as suggested by [16]: “not every *Y* needs to be affected by each confounder. However, the more *Y* each LV affects, the more information we have about it in the data, and thus the confounding proxies (i.e., LVs estimated by data) capture the effect of the confouders better”. In addition, dense assumption ensures simply tuning of the number $\hat{q}$ of confounding proxies, see a review in [41].

BAP or LV graphs consider arbitrary or pervasive patterns of confounding but we sometimes expect mixed structure. Numerous works on low-rank representation recovery have connected data manifold information in the form of a discrete graph, or its adjacency matrix, into the framework for dimensionality reduction [28, 42–45]. The fundamental hypothesis is that high-dimensional data samples are on or near a smooth low-dimensional manifold.

We propose to use as the adjacency matrix the bow-free covariance matrix that was selected by the BAP search. Specifically, let *A*(*p*, *p*) be the weighted symmetric matrix that encodes the adjacency information between the variables of dataset, *Y*(*n*, *p*) and *D* = *diag*(*d*_1_, …, *d*_*p*_) be the diagonal degree matrix with *d*_*j*_ = ∑_*k*_*A*_*jk*_. Then, $L=D^{-\frac{1}{2}}\left(D-A\right)D^{-\frac{1}{2}}$ is the definition of the normalized graph Laplacian, which describes the structure in *A*. The graph Laplacian, *L*(*p*, *p*) may be used to leverage the data manifold information in *A*, leading to different Graph Regularized PCA models. Graph Laplacian PCA (gLPCA) was introduced in this setting by [28], combining data cluster structures inherent in *A* with PCA. The model is a data representation, i.e. $\hat{Y}=PW^{T}$ where *P*(*n*, *q*) = *YW* is the projected data on the *q*-dimensional orthonormal basis, *W*(*p*, *q*) embedding the cluster structures in *A*. The spectral vectors, *W* are the eigenvectors corresponding to the first *q* smallest eigenvalues of the combined matrix:

$$\begin{array}{c} G_{\beta }=\left(1-\beta \right)\left(I_{p}-Y^{T}Y/e_{1}\right)+\beta \left(L/e_{2}+11^{T}/n\right) \end{array}$$ (13)

where *β* is a tuning parameter ∈(0, 1) weighting PCA or graph Laplacian based aspect, *e*_1_ and *e*_2_ are normalized values (see [28] for details). We use as weighted adjacency matrix the element-wise product, *A* = S*C where *S* is the covariance matrix and *C* is the unweighted adjacency matrix (1,0) of the significant bow-free covariances selected by BAP search, to extract the projected scores, *P* of gLPCA. The number of components, *q* is determined by the number of clusters by spectral clustering through cluster leading eigen() function of **igraph** R package, and the beta parameter is fixed to *β* = 0.75 or *β* = 1, if *q* > 3. In a mixed confounding regime, we suggest to add these projected scores to the input data and its uncorrelated source nodes to DAG, as in the PCA procedure.

### User interface

Any graph can have BAP deconfounding (either fitting CGGM or gLPCA) applied to it using the SEMbap() function (see help documentation: ?SEMbap). The SEMbap() pipeline employs the following R functions: Shipley.test() of **SEMgraph**, the fitConGraph() of **ggm** or the glasso() of **glasso**, and the svd() of **base** for bow-free covariance search, constrained estimation solver and spectral decomposition, respectively. The example code of the function SEMbap() is as follows.


SEMbap(graph, data, group = NULL, dalgo = "cggm",
       method = "BH", alpha = 0.05, hcount = "auto",
       cmax = NULL, limit = 200, …)


The inputs are: an igraph [46] object (*graph*); a matrix with rows corresponding to subjects and columns to graph nodes (*data*); a binary vector with 1 for cases and 0 for control subjects (*group*); the deconfounding method (*dalgo*, default = “cggm”); multiple testing correction method (*method*, default = “BH”); the significance level (*alpha*, default = 0.05); the number of latent variables (*hcount*, default = “auto); maximum number of parents set for conditional independence tests (*cmax*, default = Inf); graph size (number of nodes) switch to *glasso* for the estimation of the precision matrix (*limit*, default = 200) and other optional inputs (refer to https://rdrr.io/cran/SEMgraph/man/SEMbap for more details). We refer the reader to the Discussion section for more information about the optimal choice of the inputs of the SEMbap() function.

Both a graph and data input are required for methods involving BAP search, since the input graph will be used to recover the not-zero missing covariances. As the other methods involve SVD, only the data input is required.

Based on the deconfounding method that has been specified, SEMbap() will involve different computational steps:

- “cggm” (default) (i) BAP recovery through Shipley.test(); (ii) estimation of the constrained precision matrix, Ψ^−1^ through fitConGraph() function of ggm R package; (iii) obtain the de-correlated data matrix *Z* by multiplying the data matrix, *Y* rightward by the square root of the estimated precision matrix, $Z=Y{\hat{\Psi }}^{-1/2}$.
- “glpc”: (i) BAP recovery through Shipley.test(); (ii) fitting gLPCA and obtain confounding proxies as the last q principal component scores; (iii) extend the DAG by including these confounding proxies and add these LV scores to the data matrix, Z = cbind(P,Y).
- “pc”: (i) SVD of the observed data; (ii) first *q* principal components (projected scores) to obtain the factor scores proxies; (iii) extend the DAG by including these confounding proxies and add these LV scores to the data matrix, Z = cbind(P,Y).
- “pcss”: (i) SVD of the observed data; (ii) compute spectrum transformation matrix, *T* of singular values *d*; (iii) obtain adjusted data matrix by multiplying observed data *Y* by the spectral transformation of “pcss” method (*Z* = *TY*).

A list of four objects:

- *dag*, the DAG extracted from input graph. If (*dalgo* = “glpc” or “pc”), the DAG also includes LVs as additional source nodes.
- *guu*, the undirected graph of selected covariances; i.e, the missing edges selected after multiple testing correction. If (*dalgo* = “pc” or “pcss”), adjacency matrix is equal to NULL.
- *dsep*, the data.frame of all d-separation or CI tests over missing edges in the DAG. If (*dalgo* = “pc” or “pcss”), d-separation dataframe is equal to NULL.
- *data*, the adjusted (de-correlated) data matrix or, if (*dalgo* = “glpc” or “pc”), the combined data matrix where the first columns represent LVs scores and the other columns the raw data.

To read more about SEMbap() function, in terms of description, usage, function arguments and value, refer to https://rdrr.io/cran/SEMgraph/man/SEMbap.

### Experimental design

For testing and comparing the performance of our proposed BAP approaches with the other deconfounding methods (see Section Experimental design), we provide some experimental scenarios on synthetic data, and we evaluate the power of each method to optimally identify different structures of hidden confounding.

#### Simulation set-ups

The simulation design (4 × 6) with 100 randomization per design levels is reported in Table 1.

**Table 1:** Overview of the 4 × 6 simulation design.

		dense			sparse		
		1LV all	3LVs cluster	3LVs overlap	HDLVs sporadic	HDLVs interconnected	DAG
**p = 32**	**n = 100**	100	100	100	100	100	100
	**n = 400**	100	100	100	100	100	100
**p = 190**	**n = 100**	100	100	100	100	100	100
	**n = 400**	100	100	100	100	100	100

In detail, starting from the “Amyotrophic lateral sclerosis” (ALS) pathway from KEGG database [47], two subgraphs (see Figs A and B in S1 File). have been extracted to test for different dimensions of number of variables *p* in the simulated data. The small graph is a subgraph with 32 nodes and 47 edges whilst the larger one has 190 nodes and 259 edges. Hence, the number of variables is varied in *p* ∈ {32, 190}.

The number of samples is varied in *n* ∈ {100, 400} to test for situations of, respectively, high (*p* = 190 > *n* = 100) and low (*p* = {32, 190}<*n* = 400) dimensionality. In the former, the covariance matrix could not be semi-definite positive, preventing parameter estimates. When this occurs, the function pcor.shrink() of the corpcor R package implements the James-Stein-type shrinkage estimator, which enables covariance matrix regularization.

Based on how the few LVs affect the observed variables, two different main confounding design have been investigated: (i) dense confounding: the effect of few LVs is “spread out” over most of the observed variables, and (ii) sparse confounding: every confounding variable affects few variables in the dataset.

We consider six scenarios: three scenarios regard the dense confounding design while the remaining three the sparse confounding one. The scenarios that are considered distinguish themselves by a different structure of the error covariance matrix, Ψ the number of latent confounders, *q* and overall strength of latent confounding.

Error covariance matrix can be represented by (i) a random Factor Analysis (FA) model, Ψ = ΓΓ^*T*^+ *D*_*e*_, where Γ(*p*.*q*) is the matrix of factor loadings, ΓΓ^*T*^ represents the shared variance in the common factor structure, and the diagonal matrix *D*_*e*_ represents the specific error variances; (ii) a random uniform distributions, *U*(*min*;*max*); or (iii) a random small-word network generate by Watts-Strogats (WS) model [48] defined by dimension, neighborhood and rewired probability, *SW*(*d*, *nei*, *p*).

The diagonal entries of Ψ are defined as the sum of the mean of the absolute values of the off-diagonal elements plus a random uniform term sampled between 0.1 and 0.9. According to the chosen initial graph (small or high dimension), variances of source nodes is set to 1.

The three dense scenarios can be listed as follows:

1. **1 LV all**: *q* = 1 LV affects all the observed variables. This is a FA scenario where all the covariances are non-zero, with factor loadings sampled from an uniform distribution, *U*(0.64;0.81), respectively from medium to high loadings according to [49].
2. **3 LVs cluster**: *q* = 3 LVs affect three (not overlapping) blocks of observed variables. This is a FA scenario where three blocks of covariances are non-zero, with factor loadings sampled from an uniform distribution, *U*(0.2;0.7), respectively from low to medium loadings.
3. **3 LVs over**: *q* = 3 LVs affect three (overlapping) blocks of observed variables. This is a FA scenario where three blocks of covariances are non-zero, with factor loadings sampled from an uniform distribution, *U*(0.2;0.7), respectively from low to medium loadings, with loadings larger than 0.7 if more than one LVs affect a specific variable.
  The remaining three sparse scenarios are:
4. **High Dimensional LVs (HDLVs) sporadic**: many LVs affect sporadic (isolated) observed variables. This is a scenario characterized by hidden confounding with no modularity (no groups of the nodes that are more densely connected together than to the rest of the network) but with random affected nodes which are isolated. There are many non-zero covariances sampled from an uniform distribution, *U*(0, 1).
5. **HDLVs interconnected**: many LVs affect few interconnected modules of observed variables. This is a scenario characterized by hidden confounding with high modularity of affected nodes. There are many non-zero modules of covariances sampled from Watts-Strogats (WS) model, *SW*(*d* = *p*, *nei* = 5, *p* = 0.9).
6. **DAG**: negative control with no hidden confounding. The covariances are all 0.

**Fig 1:**
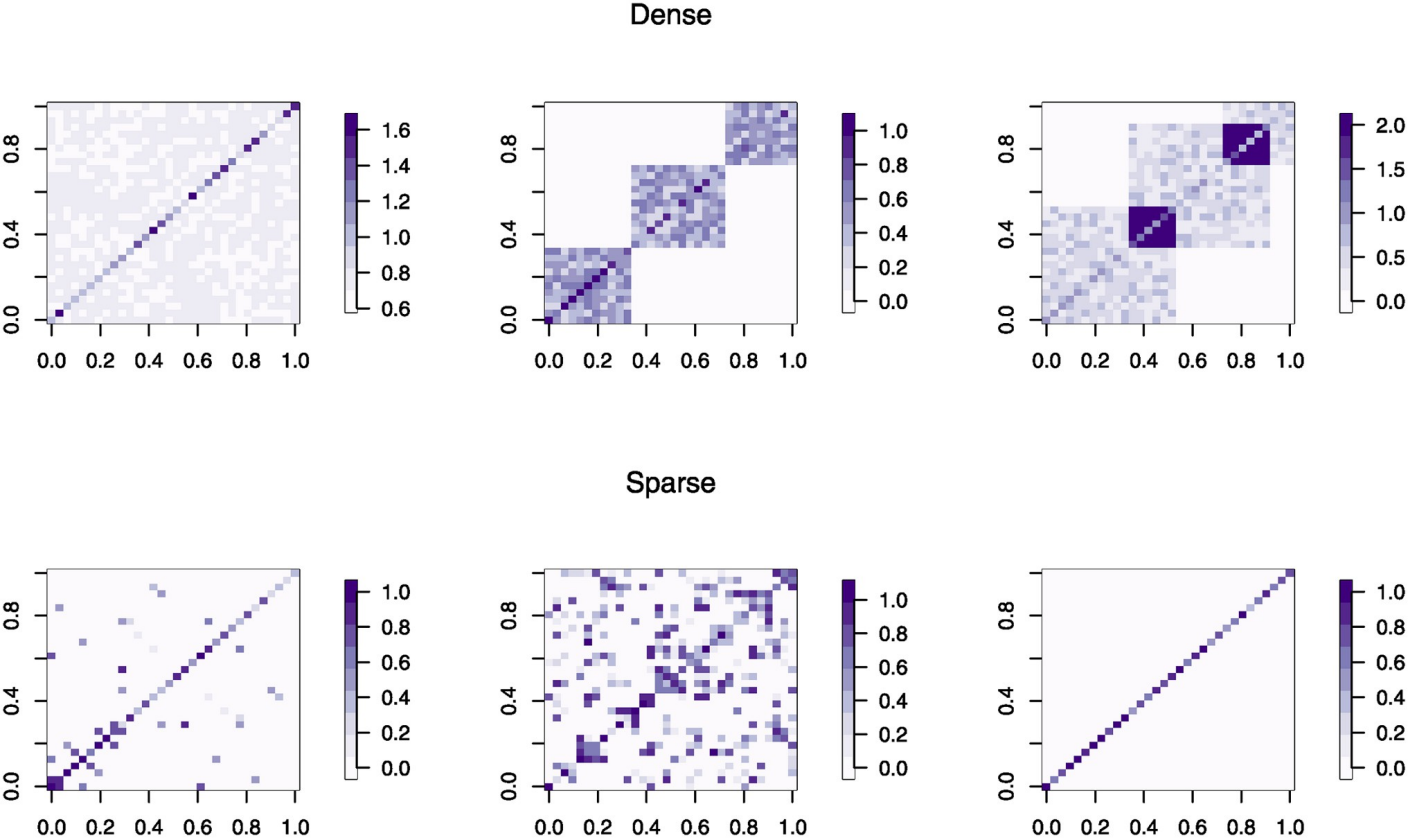
Dense and sparse simulated scenarios. Example of simulated covariance matrices with n = 400 and p = 32 for each confounding design (dense and sparse), as described in detail in Section Simulation set-ups. For dense confounding, three covariance patterns have been generated (starting from the left): (1) 1 LV all; (2) 3 LVs cluster; (3) 3 LVs over. The remaining sparse scenarios (starting from the left) are: (1) HDLVs sporadic; (2) HDLVs interconnected; (3) DAG.

Both dense and sparse scenarios can be better visualised in Fig 1.

For each of these, we generate *n* independent errors, *E*(*n*, *p*) from a multivariate normal distribution with a mean vector, *μ*(*p*, 1) and a covariance matrix, Ψ(*p*, *p*) that has an idiosyncratic component and a component due to confounding defined by the six previous scenarios.

The mean vector is sampled from an uniform distribution on the interval from 0.05 to 0.75 to recreate differential expression between cases and controls for the 25% of *p* genes, otherwise the mean vector is equal to zero.

Then, data have been generated according to *Y* = *E*(*I* − *B*)^−1^. All edge weights (i.e., the non-zero entries of the DAG coefficient matrix, *B*(*p*, *p*) of the ALS graphs are drawn from an uniform distribution on the interval between 0.1 and 1, whilst their signs are drawn from a Bernoulli distribution with probability 0.5.

#### Metrics

Varying number of samples, *n* DAG dimension, *p* and covariance matrix structure, we run 100 data simulations of each unique parameter configuration reported in Table 1, and compute the following quantities:

1. **Recovery performance measures**. Once obtained the estimated confounding covariance matrix, $\hat{\Sigma }$ for each method, we try to recover the component due to confounding in the form of adjacency matrix ([0, 1] entries) to be easily comparable with true hidden confounding matrix, with non-zero entries correspond to LVs effects. Easily for methods based on BAP search (CGGM and gLPCA), the hidden component is represented by the adjacency matrix of the BAP covariances. Differently, the other methods report the hidden component as a continuous output (being a part of the estimated covariance matrix). Specifically, the outputs obtained by each method can be listed as follows:
  - the matrix of $\hat{\Gamma }$ coefficients obtained from SEM fitting for PCA, thus the common factor covariance, $\hat{\Gamma }{\hat{\Gamma }}^{T}$;
  - the estimated dense low-rank matrix from LRpS procedure;
  - NULL covariance for SVD methods.
  These continuous matrices have been converted to binary [0, 1] format by applying a reasonable threshold to the absolute values of the confounding matrix. In the same way, the true hidden confounding matrix has been recovered from the covariance matrix of the simulated data, putting 1 for the non-zero entries. In the end, the two confounding adjacency matrices have been compared to obtain the 2x2 frequency table (i.e. confusion matrix) and the classical performance indices (precision, recall and f1-score). Let TP be the true positives, FP be the false positive, TN be the true negative, and FN be the false negative. Then, Pre = TP/(TP+FP), Rec = TP/(TP+FN), and f1-score = (2*Rec*Pre)/(Rec+Pre). The higher the metrics, the better the performance.
  In addition, (iv) false positive rate, fpr = FP/((TP+FP) has been recovered to evaluate if, in the DAG scenario with no confounding, the methods still recognize the presence of LVs. To note that for SVD methods with NULL confounding covariance, none performance metrics have been computed.
2. **Goodness-of-fit measures**. We obtain the adjusted data from each method, accounting for estimated hidden confounding. Then, we fit the ALS graph (small and large) via SEMrun() function of **SEMgraph** R package considering the unadjusted data (with hidden confounding) and the adjusted data. We obtain SEM evaluation metrics using (i) the Standardized Root Mean Square Residual (SRMR), i.e. the square root of the average of squared standardized residuals between the observed and the hypothesized covariance and the (ii) deviance/df [18], i.e. a ratio between the magnitude of *χ*^2^ and the expected value of the sample distribution *E*(*χ*^2^) = *df*. These metrics have been compared with the reference cut-off suggested from the SEM literature (0.08 − 0.10 for SRMR and 2 − 3 for deviance/df). The lower the value, the better the performance.
  In addition, it is possible to identify differentially regulated nodes (DRNs), or variables that exhibit a statistically significant difference in their activity (for example, gene expression) between the experimental and control groups, by taking into account an exogenous group variable acting over a common model. Node activation and node inhibition P-values (*P*+ and *P*−, respectively) have been combined through a Bonferroni statistics (*P* = 2*min*(*P*+; *P*−)) to obtain a measure of the ability of each method to recover group perturbation of simulated data despite confounding adjustment. As the latter statistics was transformed by the negative logarithm function (nlog10P), the higher the value, the better the performance. The ability of each method was evaluated in terms of recovery the perturbation level of not-adjusted data when removing hidden confounding. Moreover, the absolute number of nodes showing significant variation in cases with respect to healthy controls has been reported (vcountP).

#### Deconfounding methods

We refer the reader to the S1 File for a brief overview about the alternative competitors of CGGM and gLPCA, based only on a pervasive confounding assumption. Specifically, we consider procedures based on spectral transformation [10], and Low Rank plus Sparse model [13]. Table 2 provides an overview of the deconfounding methods in terms of type of algorithm employed, input requirements, confounding assumption and methodological steps together with main papers for reference.

**Table 2:** Overview of the considered deconfounding methods.

Method	Reference	Algorithm	Input data	Confounding assumption	BAP search	SVD
CGGM	[27]	Constrained Gaussian Graphical Model (w/ dsep search)	Gene expression and graph object	Arbitrary	Yes	No
gLPCA	[28]	Graph-Laplacian PCA (w/ dsep search)	Gene expression and graph object	Mixed	Yes	No
LRpS	[13]	Low rank plus sparse decomposition	Gene expression	Dense	No	No
PCA	[16]	Singular Value Decomposition (SVD)	Gene expression	Dense	No	Yes
PCSS	[50]	Spectral trasformation	Gene expression	Dense	No	Yes
Trim	[10]	Spectral trasformation	Gene expression	Dense	No	Yes

Besides the type of algorithm, these methods differ in two main aspects: (i) the input requirements, gene expression data and graph object or only the former; (ii) the confounding assumption; (iii) methodological steps, BAP search or SVD. Unlike the other methods, CGGM, and gLPCA requires as input also a graph object, since CGGM and gLPCA algorithm involves BAP search with d-separation tests or CI tests between all pairs of DAG missing edges.

The remaining methods only require a gene expression data as input since their approach involves a SVD on observed data or the ADMM as for LRpS algorithm. Most of the methods work under the structural assumptions regarding the sparsity of the underlying DAG and the denseness of latent effect, except for CGGM, and gLPCA, where the confounding assumption is arbitrary or mixed. In detail, the former refers to an arbitrary hidden structure with few or many sporadic LVs and the latter to a mixed hidden structure with few pervasive LVs defined by cluster structures.

As a result, since different experimental designs have been tested within simulation runs (see Section Experimental design), some methods are expected to perform better than others depending on the starting confounding assumption. Hence, the goal is to find a deconfounding method that represents an optimal solution in both situations.

## Results

In this section, we evaluate the performance of our proposed approaches against the other state-of-the-art deconfounding methods both on simulated and observed expression data to provide an efficient solution for different confounding scenarios.

### Simulation results

The relative performance of all methods has been summarized under different experimental conditions on 100 simulation replications to better quantify the efficiency of each deconfounding method against our BAP search approach (CGGM and gLPCA).

Some preliminary considerations need to be made before discussing simulation results:

- Given that SVD methods report NULL covariance, it is not possible to recover performance measures in terms of hidden confounding; in this case, the results will be referred only to CGGM, gLPCA, LRpS and PCA.
- Since our approaches share the same first stage of BAP search, the hidden component is represented by the same adjacency matrix of the BAP covariances. As a result, covariance recovery performance measures for CGGM and gLPCA have been aggregated in CGGM/gLPCA.
- Obviously, already existing methods are expected to perform better in the dense confounding designs, given their fully pervasive confounding assumption.
- Note that the results referring to the case with *n* = 400, since in this case we obtain more robust evaluation metrics. In some cases, when *n* < *p*, it could happen that matrix regularization generates a near identity matrix and, as a result, misleading evaluation metrics. We refer the reader to the S1 File for all the results regarding the experimental design with *n* = 100 and additional results for *n* = 400.

#### Recovery performance measures

**Fig 2:**
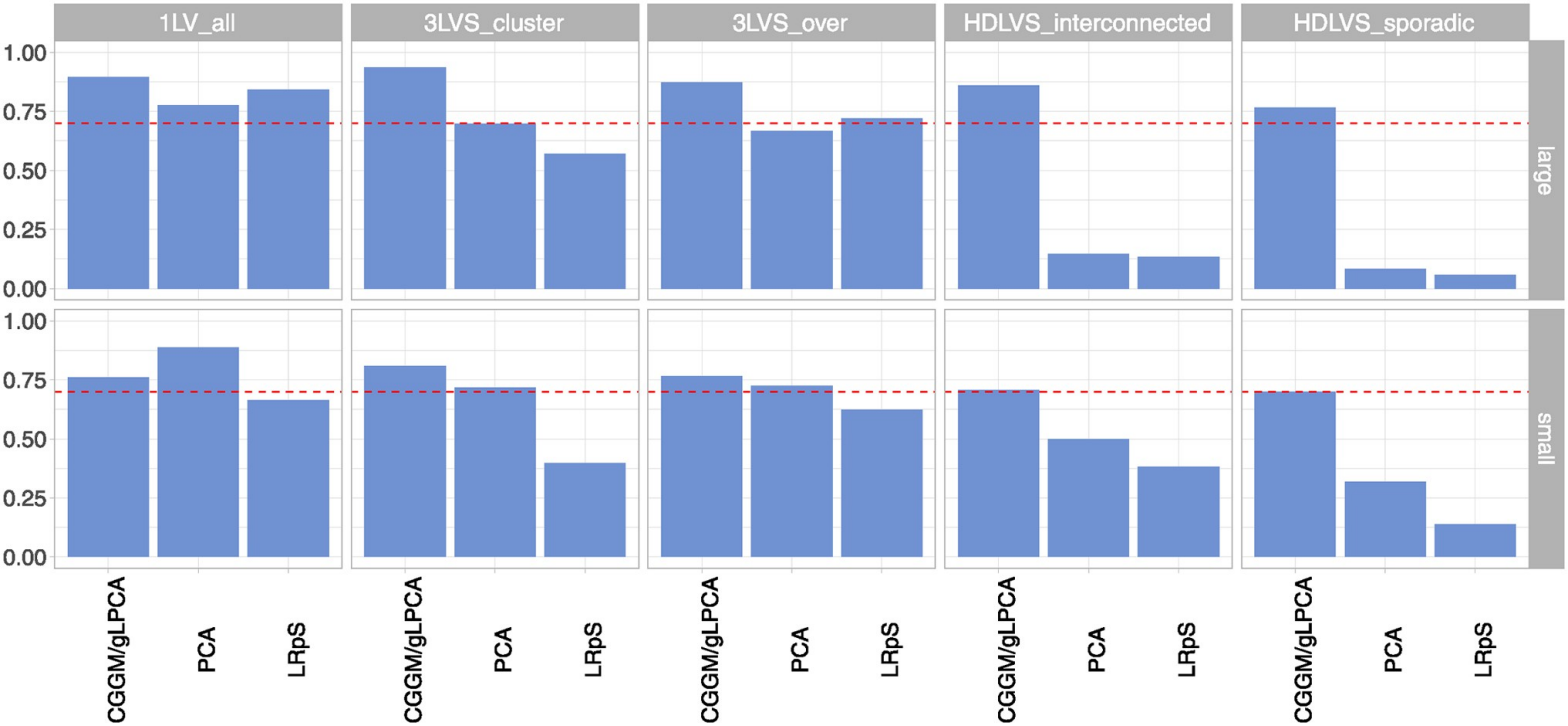
F1-score for simulated data. F1-score summarised as mean over simulations for dense/sparse confounding design with n = 400. For SVD methods with NULL confounding covariance, none performance metrics have been computed.

Fig 2 shows the f1-score summarised as mean over simulations for pervasive (1*LV*_*all*, 3*LVS*_*cluster*, 3*LVS*_*over*) and arbitrary (*HDLVs*_*interconnected*, *HDLVs*_*sporadic*) confounding design with *n* = 400.

Fig 2 reports high recovery performance metrics for our BAP-based methods (CGGM/gLPCA), reporting an f1-score around 0.9 for the large graph and around 0.75 for the small graph scenario in the dense confounding case. In almost all sparse confounding scenarios, the methods report around 0.8 for the large graph and 0.7 for the small graph, with a maximum of 0.9 for the *HDLVS*_*interconnected* design. As reported in Table A in S1 File, in the dense confounding case, CGGM/gLPCA recovers both an high proportion of covariances compared to the true ones (recall between 0.7 and 0.8) and an high number of correctly identified covariances over the estimated ones (precision between 0.85 and 1), thus allowing to correctly identify most of the hidden confounding in the different simulated scenarios. In addition, Table B in S1 File shows if the methods are able to control fpr in the DAG scenario with no hidden confounding. CGGM/gLPCA show a fpr almost equal to 0.

PCA seems to reach the level of (approximately) 0.7 f1-score for all the dense confounding scenarios, except for 1*LV*_*all* design in the small graph case where the method exceeds the threshold of 0.7, reaching an f1-score of 0.9. As expected, in this sparse scenario, PCA reports a f1-score around (or below) 0.15 for the large graph case; the latter method is able to reach the 0.5 threshold only in the *HDLVS*_*interconnected* design with regards to the small graph case (Fig 2). PCA is able to control the error rate, reporting a fpr around 0.1 (Table B in S1 File).

LRpS reports an high f1-score (around 0.7–0.8) for 1*LV*_*all* design and lower metrics for the 3*LVs*_*cluster* scenario. For the sparse scenarios, almost same conclusions as for PCA can be reported for LRpS (Fig 2). Generally, as shown in Table A in S1 File, in most of the sparse cases, the precision levels were really low. To note that Table B in S1 File shows that the largest proportion of false hidden confounding is recovered by LRpS for the largest graph case (0.313).

In conclusion, CGGM/gLPCA methods are able to obtain higher recovery performance measures in all confounding scenarios, unlike the other methods that correctly (as expected) identify confounding only in some of the dense confounding scenarios, thus reporting higher error rate in the other cases.

#### Goodness-of-fit measures

A good performance is also characterized by a low SRMR value together with a good perturbation level ((nlog10P and vcountP). Tables C and D in S1 File show the SRMR and perturbation scores for each method summarized as mean across simulations, respectively for arbitrary and dense confounding design.

CGGM reports low values around 0.1 for most of the cases whilst gLPCA has higher SRMR score for the dense confounding case, given that, to prevent overfitting when the number of identified clusters in the recovered subnetwork is higher than 3, the gLPCA procedure switches to full Laplacian graph.

The lowest values of SRMR are reported by PCA in almost all sparse and dense confounding scenarios (around 0.03). The worst SRMR score, around 0.2, is reported by PCSS in both the dense and sparse confounding design (small graph case).

Even after accounting for hidden confounding, nearly all of the approaches can recover the same amount of perturbation of the simulated data (nlog10P and vcountP). For the dense confounding scenarios, only LRpS is unable to recover sufficient perturbation metrics.

Thus, in the end, CGGM/gLPCA and PCA procedures are able to recover higher goodness-of-fit measures compared to the alternative approaches, while retaining most of the original data perturbation.

For more details about simulation results, we refer the S1 File.

### Application to real genomic data

#### BRCA RNA-seq data

We make use of the breast cancer (BRCA) RNA transcriptomics profiling data from TCGA [51] that has been downloaded with the curatedTCGAData [52] R-package. The data matrix has *p* = 19247 genes and *n* = 224 human samples, consisting of 112 primary solid tumor samples matched with 112 solid tissue normal samples. The data have been normalized using nonparanormal transformation [53]. As a *priori* graph, we use the “Breast Cancer Pathway” (*hsa05224*) from KEGG which contains 133 nodes and 483 edges. The latter graph has been mapped on BRCA data, resulting in a set of 131 nodes (i.e., selected genes in the data matrix) and 481 edges.

We follow the approach of [50] to evaluate how the methods handle hidden confounding by eliminating the transcription factors (TFs), i.e., we create confounders by design. Specifically, public databases like TRRUST [54], which are accessible to the general public, give lists of transcription factors (TFs) together with the genes (referred to as targets) that they regulate.

TFs list is frequently discussed in the literature because it serves as an illustration of a real-world data set for which the existence and orientation of some edges is known, allowing for the comparison of estimated graphs with a “partial ground truth” [55, 56]. Because TFs are so important in the genesis of cancer, it is thought that manipulating the expression of certain genes could change the course of specific tumors. There is agreement regarding the significance of some transcription factor families in the genesis of cancer [57, 58].

In detail, there are 22 TFs from TRRUST that map 131 genes from the breast cancer graph. We remove all 22 TFs from the graph and assume that we only observed the remaining 109 genes, i.e we analyzed a data set of dimension (224 x 109). The minimal sparsity index has been estimated equal to 3, meaning that in sparsity condition every node is connected to at maximum 3 nodes, while in the BRCA graph the mean of connected node is 4. The number of degree of freedom for BAP search, i.e. the number of missing edges to be tested in the BRCA graph, is 5849.

#### BRCA RNA-seq results

Before running the analysis, the number of confounding proxies for PCA deconfounding methods (i.e. PCA and PCSS) has been specified. As described in the S1 File, the number of LVs has been determined according to a permutation method and the scree plot has been visualized where eigenvalues are displayed against the number of the principal component. The number of confounding proxies selected by the permutation method is 7, resulting in 55% of explained total variance. However, we’ve reduce the number of LVs from a maximum of 7 to an optimal number of 3, that explains 41% of total variance, based on a trade-off between SEM fitting and perturbation metrics. The number of LVs in gLPCA is defined in the cluster information encoded in graph data equal to 3.

A good performance was detected if, after removing hidden confounding, (i) SRMR (or dev/df) was below the not-adjusted level and the 0.08 (or 2) threshold value; (ii) perturbation level (nlog10P or vcountP) approximates the not-adjusted value. Thus, we want to evaluate if the methods are able to adjust the data while retaining most of data perturbation. See Table 3 for benchmark results.

**Table 3:** Evaluation metrics (SRMR, dev/df, nlog10P and vcountP) from benchmark data analysis.

Method	SRMR	dev/df	nlog10P	vcountP
Unadjusted	0.151	3.565	13.767	85
CGGM	0.085	2.446	12.583	55
gLPCA	0.083	2.639	13.628	71
LRpS	0.02	0.104	0.068	5
PCA	0.078	2.296	11.897	69
PCSS	0.128	7.023	2.702	6
Trim	0.045	0.895	3.136	35

Regarding our BAP search approach, gLPCA reports a slightly higher SRMR (0.083) and is able to recover almost all data perturbation (nlog10P = 13.628 and vcountP = 71) over respectively 13.767 and 85 for the unadjusted data. Also CGGM reports a good performance but with a lower number of differentially regulated nodes (55).

PCA reaches the lowest SRMR value (0.078) while retaining a perturbation level (11.897) with 69 differentially regulated nodes. LRpS and Trim recover the lowest SRMR values (respectively 0.02 and 0.045) but has also the lowest level of retained perturbation. Thus, these methods aggressively adjusts the data while losing a huge portion of information. The SRMR value could be a sign of overfitting problems. PCSS shows a bad SRMR value (0.128) above the 0.08 threshold and really low perturbation metrics (nlog10P = 2.702 and vcountP = 6).

**Fig 3:**
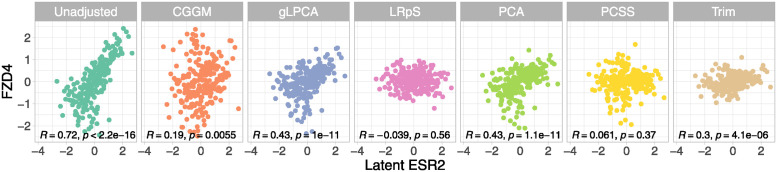
Benchmark data decorrelation (Transcription factor ESR2 vs Gene FZD4). Gene FZD4 has a positive correlation greater than 0.5 with the unobserved transcription factor ESR2 (Unadjusted). After subtracting out the confounding variation estimated using the different methods for each gene (denoted as “deconfounded” expression level), the genes are no longer correlated with ESR2.

**Fig 4:**
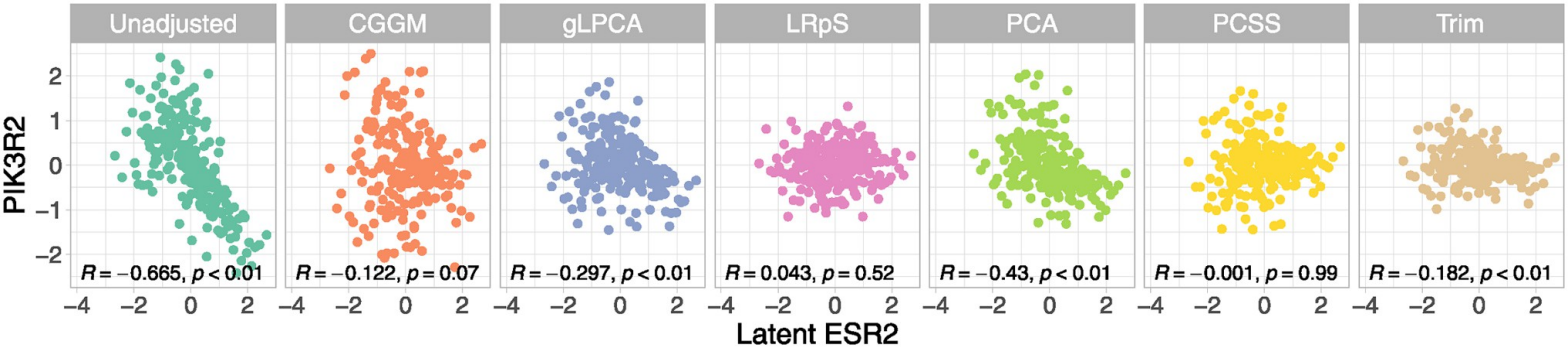
Benchmark data decorrelation (Transcription factor ESR2 vs Gene PIK3R2). Gene PIK3R2 has a negative correlation greater than 0.5 with the unobserved transcription factor ESR2 (Unadjusted). After subtracting out the confounding variation estimated using the different methods for each gene (denoted as “deconfounded” expression level), the genes are no longer correlated with ESR2.

We can also assess how well the techniques deal with hidden confounding, as we have removed the TFs but we know their true values. In Figs 3 and 4, we examine the highest positively and negatively linked genes with the transcription factor ESR2, i.e. FZD4 and PIK3R2, respectively. In addition, the figures report the Pearson’s correlation coefficients (*R*) with their p-values (*p*) related to the correlation of FZD4 and PIK3R2 with ESR2. As can be seen in the first box, the genes’ unadjusted expression level correlates well with ESR2, with a significant positive correlation between FZD4 and ESR2 (*R* = 0.72, *p* = 7.44*e*^−37^) and a significant negative correlation between PIK3R2 and ESR2 (*R* = −0.665, *p* = 2.18*e*^−30^). In the other boxes, we observe how the shared confounding effect of the transcription factor ESR2 is removed using the various deconfounding techniques. Specifically, we want to evaluate if the other methods are able to lower the R correlation coefficient of the original (unadjusted data). CGGM is able to lower significantly the original correlation level, with a *R* = 0.19 (*p* = 0.006) and *R* = −0.122 (*p* = 0.07) for, respectively, the FZD4-ESR2 and PIK3R2-ESR2 coefficients. In both cases, the magnitude reflects a negligible (almost null) correlation between the genes. gLPCA and PCA report the same results in the FZD4 case, being able to lower the correlation less than CGGM (*R* = 0.434, *p* = 1*e*^−11^); however, in the ESR2 case, gLPCA reports a lower correlation coefficient (*R* = −0.297, *p* = 5.97*e*^−06^) than PCA (*R* = 0.43, *p* = 1.07*e*^−11^). As expected, LRpS and PCSS report an almost null correlation coefficient, reflecting their aggressive adjustment of the data without retaining the original data perturbation (Table 3). Trim reports a good ability to lower the correlation coefficient, but without the support of a good performance in terms of data perturbation in Table 3.

We can conclude that CGGM and gLPCA, compared to the other methods, are able to adjust more effectively the BRCA data for hidden confounding, being able to lower the correlation with the TFs while keeping a high proportion of data perturbation.

Furthermore, the reader can refer to the S1 File to better visualise the starting subnetwork of the benchmark data analysis together with the recovered ones from the d-separation tests’ procedure. In this way, the arbitrary confounding design is displayed, with many LVs affecting observed ones.

In detail, Fig C in S1 File displays the “Breast Cancer Pathway” (including highlighted TFs and colored level of perturbation), representing the starting graph of SEMbap() algorithm. Fig D in S1 File shows the subnetwork of adjacency matrix obtained from d-separation tests of SEMbap() algorithm, adding yellow nodes representing LVs to the latter subnetwork.

## Discussion

We have discussed the problem of dealing with unobserved confounding factors to correctly quantify interesting biological signals. Building on existing literature [9, 10, 13], a two-stage (deconfounding plus fitting) procedure based on Bow-free Acyclic Paths (BAP) search developed into the framework of SEM has been proposed. The existing deconfounding methods differ in the way they perform the first stage, i.e.:

- directly estimate confounding variables from the data as the scores of the first *q* principal components and simply add them to the data matrix, creating an augmented data matrix;
- transform data by applying a linear transformation that only transform the singular values of the data, while keeping it singular vectors intact;
- decompose the concentration matrix as a sum of a sparse matrix and a low-rank matrix where the latter reveals the number and effect of the hidden variables.

Instead, our approach first makes an exhaustive BAP search of missing edges with significant covariance with Shipley’s independent d-separation or CI local tests and then either (i) fit the inverse of the selected covariance matrix via CGGM and decorrelate the data matrix via Mahalanobis’s trasformation or (ii) learn a low dimensional representation of the observed data matrix that incorporates graph structures and add the last *q* principal component scores to the data matrix.

After removing hidden confounding, based on the goal of the analysis, the methods can perform a second stage where the modified data can be used as an input for SEM fitting, a high-dimensional sparse regression technique or for any structure learning algorithm. Since our approach starts from a knowledge-based biological network (i.e., either ALS or BRCA provided by KEGG database, in our simulated or real data examples), we aim to adjust the data for hidden confounding, map the adjusted data matrix onto the input graph and convert it into a SEM to assess goodness-of-fit (SRMR, dev/df) and perturbation recovery (nlog10P, vcountP).

Based on the results obtained from both the simulated and real data analysis, we can make some considerations to facilitate the user’s choice of the input method within the algorithm.

Our methodology (CGGM, glPCA) differs from the other methods since it requires a priori graphical structure as input and makes use of both arbitrary (CGGM), or mixed (gLPCA) deconfounding assumption (based on the chosen combination of methodological steps).

Simulation results report an outstanding performance of CGGM and gLPCA in both sparse and dense confounding scenarios. In benchmark data analysis, best performances are reported by gLPCA, immediately followed by CGGM. However, gLPCA can be preferred over CGGM methodology because the former methods add the first (or last) principal components as additional source nodes without adjusting the existing data matrix.

Unlike our methods, the other approaches operate only under the structural hypotheses of the density of latent effect. Thus, the other methods show acceptable performance only in the dense confounding scenarios of the simulated data. In some cases, PCA represents a viable algorithm and is able to recover a performance near our approach. The real data analysis allows to confirm the efficiency of PCA algorithm and highlights the worst performances of PCSS and LRpS.

We have provided three different optimal choices that can be used by the reader based on its needs. PCA represents an efficient algorithm in case of dense confounding, whilst CGGM and gLPCA can be implemented in case of sparse confounding or a mixture or both.

Lastly, the reader needs to be aware that, to obtain an optimal performance of the deconfounding methodology, the inputs of BAP search algorithms need to be properly tuned, especially with respect to:

- *alpha* (default = 0.05): False discovery rate (FDR) significance level for Shipley’s local d-separation tests. The data de-correlation process is controlled by this argument. Table 4 reports a sensitivity analysis of the proposed methods (CGGM, glPCA) on the benchmark data analysis with different alpha levels. Since there are no significant oscillations in the results, it could be stated that the method is robust to different choices of the alpha level. To note that the data de-correlation process is not enabled if alpha = 0.
- *cmax* (default = NULL): maximum number of parents set. In more detail, this option can only be applied to run tests where the number of conditioning variables does not go over the specified value. Conditional independence tests with a high dimensionality may not be very reliable. Our recommendation is to test bow-free covariances with basis set sizes close to the sparsity index, $s=\sqrt{n}/log\left(p\right)$, to drive the sparsity. Alternatively, the user can switch to the glasso procedure if the graph size is huge by setting the limit argument (default = 200). To note that this input is needed only for BAP search methods, i.e. “cggm” and “glpc”.
- *hcount* (default = “auto”): the number of latent (or hidden) variables. This input is needed only for the PCA method (dalgo = “pc”). By default hcount=“auto”, the hidden count is determined with a permutation approach where, permuting the columns of the data matrix, Y, the singular values are compared to what they would be if the variables were independent, and components are chosen if their singular values are greater than those of the permuted data (for a review see [59]). To note that in the “glpc” case, the number of hidden variables is equal to the number of clusters of the identified BAP covariances.

**Table 4:** Sensitivity analysis of the alpha level of the SEMbap() function.

method	alpha	srmr	dev_df	nlog10P	vcountP
CGGM	0.1	0.083	2.388	12.200	52
	0.05	0.085	2.446	12.583	55
	0.005	0.092	2.640	12.708	57
	0.001	0.097	2.730	12.384	58
gLPCA	0.1	0.093	1.922	13.649	78
	0.05	0.074	1.690	13.628	71
	0.005	0.074	1.688	13.628	70
	0.001	0.074	1.686	13.628	69

Further studies could look at combining the deconfounding problem with causal discovery algorithms [22], allowing the user to use the proposed deconfounding approach not only starting from a priori knowledge-based network, but also from a fully data-driven network. Moreover, once the hidden confounding has been removed from the extracted graph, a successive data-driven network will be recovered to represent true data variation.

In conclusion, we have shown that SEMbap() is easily accessible to users and provides several methods to deal with hidden confounding under several assumptions. We have introduced and validated (both on simulated and real data) a two-stage deconfounding plus fitting procedure based on BAP search. Results report that CGGM and gLPCA are able to correctly identify hidden confounding whilst controlling false positive rate and achieving good fitting and perturbation metrics in both sparse and dense confounding scenarios. We believe that, both CGGM and gLPCA can valuable tools for practitioners when undertaking complex sparse confounding scenario, while PCA can be used in case of pervasive (dense) confounding.

## Supporting information

S1 File Additional material about data analysis. This PDF file contains two sections with additional information, tables and figures.
(PDF)
