# Supplementary material for: SEMbap: Bow-free covariance search and data de-correlation
Source: PLoS Comput Biol. 2024 Sep 11;20(9):e1012448. doi: 10.1371/journal.pcbi.1012448 (PMC11419354; doi:10.1371/journal.pcbi.1012448)
Supplement: S1 File — This PDF file contains two sections with additional information, tables and figures. (PDF) [file pcbi.1012448.s001.pdf]

Supplementary material for "SEMbap: Bow-free covariance search and data de-correlation" by Grassi M and Tarantino B

Contents

1 Alternative procedures **1**

2 Additional results **2**

List of tables

A Precision, recall and F1 score summarised as mean over simulations for dense/sparse confounding design with n=400. 2

B False positive rate summarised as mean over simulations for sparse confounding design (DAG) with n=400 and n=100. 3

C Evaluation metrics (srmr and nlog10(P)) summarised as mean over simulations for arbitrary confounding design with n=400. 4

D Evaluation metrics (srmr and nlog10(P)) summarised as mean over simulations for dense confounding design with n=400. 5

E Precision, recall and F1 score summarised as mean over simulations for dense/sparse confounding design with n=100. 6

F Evaluation metrics (srmr and nlog10(P)) summarised as mean over simulations for arbitrary confounding design with n=100. 7

G Evaluation metrics (srmr and nlog10(P)) summarised as mean over simulations for dense confounding design with n=100. 8

List of figures

A Small subgraph from ALS pathway for simulated data (32 nodes and 47 edges). 9

B Large subgraph from ALS pathway for simulated data (190 nodes and 259 edges). 9

C Perturbed original subnetwork from benchmark data analysis. Nodes in the recovered subnetworks are coloured in yellow if they represent TFs, pink-shaded if significantly activated or blue-shaded if signficantly inhibited. 10

D Subnetwork of adjacency matrix obtained from d-separation tests of SEMbap() algorithm with yellow nodes corresponding to LVs in benchmark data analysis. 10

E F1-score summarised as mean over simulations for dense/sparse confounding design with n=100. For SVD methods with NULL confounding covariance, none performance metrics have been computed. 11

1 Alternative procedures

Alternative competitors to our BAP+CGGM and BAP+gLPCA are proposed in literature only for pervasive confounding. Specifically, we consider procedures based on spectral transformation, and Low Rank plus Sparse model.

Spectral transformation. The idea of spectral transformation is to transform data, Y by applying a linear transformation, TY that only transform the singular values of the data, while keeping it singular vectors intact. It is designed to reduce the magnitude of dense confounding. In particular, when the LVs aligns with the top singular values of Y (i.e. the magnitude of LV effects are large compared with specific error terms), a SEM is designed on the transformed data (in matrix form):

$$TY = TYB^T + U, \text{ with } \text{cov}(U) = D_\psi \tag{1}$$

making standard DAG assumptions. Here, we look at the "trim" and "pcss" transformations implemented with hidden routines of `SEMbap()` function of **SEMgraph** R package.

Trimm method. The trimm method upper-bounds each singular value to $d_m := median(d_1, ..., d_r)$, and the spectral transformation is given by $T := PD_mP^T$, where D_m is diagonal with each element on the diagonal equal to $D_m(ii) := min(d_i; d_m)/d_i$. Therefore:

$$Z = TY = PD_mP^TY = (PD_mP^T)(PDQ^T) = PD_tQ^T \tag{2}$$

where $D_t(ii) := min(d_i; d_m)$. The spectral transformation keeps the ordering of singular values in the transformed design matrix while still shrinking the large ones, and an advantage is that we do not have to estimate the number of LVs. See (1) for additional details.

PCSS method. PCSS consider the commonly used approach to extract the first $q < r$ principal component of Y. The principal components (or the projected scores), $P(n, q)$ serve as a measuring proxies for LVs; i.e., are "sufficient statistics" (pcss) for unobserved scores, if the confounders are dense or pervasive. Ideally q is equal to or slightly larger than the dimension of dense confounders. One then adjusts data by using the partial residuals after a linear regression of Y on P:

$$Z = Y - P\hat{B} = Y - P(P^TP)^{-1}P^TY = (I_p - H)Y \tag{3}$$

where $H = P(P^TP)^{-1}P^T$ is the projection matrix on the principal component space. These partial residuals can be interpreted in term of a spectral transformation, TY where $T = PD_0P^T$ with $D_0 = diag(0_1, .., , 0_q, 1_{q+1}, ..., 1_r)$,; i.e. the first q singular values are set to zero, and the others remain intact. Of course we have that $T = I_p - H$. See (2) for additional details.

Low Rank plus Sparse model. In a pervasive regime, Low Rank plus Sparse (LRpS) (3) of the GGM can be applied. LRpS learns a observed precision matrix assuming that variables are partially observed, $Y = (Y_O, Y_H)$ and the precision matrix is partitioned as:

$$\Sigma^{-1} = \begin{pmatrix} K_O & K_{OH} \\ K_{HO} & K_H \end{pmatrix} \tag{4}$$

The matrix, K_O describes the estimated precision matrix between p observed variables, the matrix, K_H represents the estimated effect of q hidden(latent) variables, and K_{OH} =cov($Y_O; Y_H$). By standard theory for multivariate Gaussian distributions, the conditional covariance matrix of $Y_O|Y_H$ is:

$$\Sigma_O^{-1} = K_O - K_{OH}K_H^{-1}K_{HO} = K_O - L \tag{5}$$

For sparse DAGs, K_O is a sparse matrix and for $q << p$, L is low-rank with rank(L) = q. The K_O is the target matrix for inference. Hence, when the confounders are pervasive, it is possible to estimate each

component through a low-rank plus sparse matrix decomposition using the Alternating Direction Method of Multipliers (ADMM) solver applied to a covariance matrix in stage 1, and using the estimated covariance, $\hat{K}_O^{-1}$ in stage 2.

We use the `lrpsadmm()` function of **lrspadmm** R package to compute ADMM, and random normal multivariate data from the estimated sparse matrix, $\hat{K}_O$ as de-correlated data, $Z =$rnorm$(\mu = 0, \Sigma = \hat{K}_0^{-1})$ in SEM fitting, following the two-stage procedure suggested by (4).

2 Additional results

The relative performance of all methods has been summarized under different experimental conditions on 100 simulation replications to better quantify the efficiency of each deconfounding method. The results regarding the experimental design with $n = 400$ and $n = 100$ are presented below. However, in the main paper, only the case with $n = 400$ has been discussed, since in this case we obtained more robust evaluation metrics.

Obviously, some methods are expected to perform better than others in some experimental set-ups based on their confounding assumption (i.e. arbitrary or dense). As the recovered adjacency matrix representing hidden confounding is the same, classification metrics for CGGM and gLPCA have been aggregated.

Table A: Precision, recall and F1 score summarised as mean over simulations for dense/sparse confounding design with n=400.

**method**	**design**	**graph**	**precision**	**recall**	**f1**
CGGM	1LV_all	large	1.000	0.814	0.895
		small	1.000	0.616	0.760
	3LVS_cluster	large	0.996	0.882	0.935
		small	0.918	0.724	0.808
	3LVS_over	large	0.996	0.776	0.872
		small	0.966	0.636	0.766
	HDLVS_interconnected	large	0.947	0.786	0.859
		small	0.694	0.720	0.706
	HDLVS_sporadic	large	0.835	0.652	0.766
		small	0.658	0.758	0.700
LRpS	1LV_all	large	1.000	0.727	0.842
		small	1.000	0.502	0.663
	3LVS_cluster	large	0.431	0.846	0.571
		small	0.331	0.510	0.397
	3LVS_over	large	0.620	0.856	0.719
		small	0.633	0.621	0.624
	HDLVS_interconnected	large	0.072	0.860	0.133
		small	0.292	0.561	0.382
	HDLVS_sporadic	large	0.030	0.756	0.058
		small	0.085	0.405	0.139
PCA	1LV_all	large	1.000	0.638	0.777
		small	1.000	0.803	0.888
	3LVS_cluster	large	0.730	0.674	0.698
		small	0.654	0.805	0.718
	3LVS_over	large	0.865	0.545	0.668
		small	0.822	0.652	0.724
	HDLVS_interconnected	large	0.127	0.186	0.148
		small	0.460	0.547	0.498
	HDLVS_sporadic	large	0.054	0.183	0.082
		small	0.223	0.571	0.318

Table B: False positive rate summarised as mean over simulations for sparse confounding design (DAG) with n=400 and n=100.

**n**	**design**	**method**	**graph**	**fpr**
400	DAG	CGGM	large	1.40E-04
			small	3.91E-05
		LRpS	large	0.313
			small	0.005
		PCA	large	0.064
			small	0.136
100	DAG	CGGM	large	0.00E+00
			small	1.95E-05
		LRpS	large	0.624
			small	0.034
		PCA	large	0.038
			small	0.065

Table C: Evaluation metrics (srmr and nlog10(P)) summarised as mean over simulations for arbitrary confounding design with n=400.

**method**	**design**	**graph**	**srmr**	**nlog10P**
sim	DAG	large	0.041	12.655
		small	0.042	9.911
	HDLVS_interconnected	large	0.076	11.611
		small	0.171	6.538
	HDLVS_sporadic	large	0.070	12.343
		small	0.129	8.630
CGGM	DAG	large	0.041	12.779
		small	0.042	9.655
	HDLVS_interconnected	large	0.087	12.467
		small	0.137	11.567
	HDLVS_sporadic	large	0.098	12.730
		small	0.107	11.127
gLPCA	DAG	large	0.041	12.671
		small	0.042	9.978
	HDLVS_interconnected	large	0.065	11.471
		small	0.132	7.747
	HDLVS_sporadic	large	0.060	12.344
		small	0.106	9.561
LRpS	DAG	large	0.009	0.471
		small	0.040	0.089
	HDLVS_interconnected	large	0.066	0.941
		small	0.178	0.261
	HDLVS_sporadic	large	0.056	0.722
		small	0.130	0.191
PCA	DAG	large	0.030	12.504
		small	0.027	10.308
	HDLVS_interconnected	large	0.037	12.374
		small	0.044	11.269
	HDLVS_sporadic	large	0.039	12.543
		small	0.039	11.272
PCSS	DAG	large	0.018	1.962
		small	0.103	5.496
	HDLVS_interconnected	large	0.080	7.709
		small	0.262	11.883
	HDLVS_sporadic	large	0.069	5.375
		small	0.210	11.091
Trim	DAG	large	0.029	7.933
		small	0.043	8.133
	HDLVS_interconnected	large	0.026	12.611
		small	0.108	10.628
	HDLVS_sporadic	large	0.019	12.591
		small	0.085	9.579

Table D: Evaluation metrics (srmr and nlog10(P)) summarised as mean over simulations for dense confounding design with n=400.

**method**	**design**	**graph**	**srmr**	**nlog10P**
sim	1LV_all	large	0.341	10.992
		small	0.435	6.713
	3LVS_cluster	large	0.213	12.743
		small	0.239	7.617
	3LVS_over	large	0.224	11.618
		small	0.283	6.111
CGGM	1LV_all	large	0.075	11.068
		small	0.083	11.522
	3LVS_cluster	large	0.072	12.032
		small	0.109	11.773
	3LVS_over	large	0.076	11.012
		small	0.144	12.451
gLPCA	1LV_all	large	0.085	9.976
		small	0.060	5.443
	3LVS_cluster	large	0.128	12.958
		small	0.147	9.172
	3LVS_over	large	0.154	12.058
		small	0.179	8.336
LRpS	1LV_all	large	0.006	0.443
		small	0.094	0.153
	3LVS_cluster	large	0.022	0.523
		small	0.192	0.150
	3LVS_over	large	0.012	0.490
		small	0.198	0.168
PCA	1LV_all	large	0.031	8.111
		small	0.036	6.472
	3LVS_cluster	large	0.034	13.214
		small	0.039	8.648
	3LVS_over	large	0.030	12.690
		small	0.040	10.137
PCSS	1LV_all	large	0.063	9.866
		small	0.141	11.688
	3LVS_cluster	large	0.082	10.418
		small	0.209	10.985
	3LVS_over	large	0.095	10.704
		small	0.248	11.780
Trim	1LV_all	large	0.015	10.850
		small	0.063	10.702
	3LVS_cluster	large	0.031	11.968
		small	0.095	10.047
	3LVS_over	large	0.045	11.525
		small	0.133	11.132

Table E: Precision, recall and F1 score summarised as mean over simulations for dense/sparse confounding design with n=100.

method	design	graph	precision	recall	f1
CGGM	1LV_all	large	1.000	0.198	0.316
		small	1.000	0.349	0.513
	3LVS_cluster	large	1.000	0.436	0.605
		small	0.975	0.565	0.713
	3LVS_over	large	1.000	0.198	0.331
		small	0.986	0.391	0.558
	HDLVS_interconnected	large	0.994	0.277	0.433
		small	0.836	0.536	0.652
	HDLVS_sporadic	large	0.986	0.480	0.645
		small	0.792	0.631	0.698
LRpS	1LV_all	large	1.000	0.704	0.827
		small	1.000	0.552	0.709
	3LVS_cluster	large	0.378	0.803	0.514
		small	0.342	0.426	0.374
	3LVS_over	large	0.595	0.771	0.672
		small	0.637	0.529	0.575
	HDLVS_interconnected	large	0.073	0.898	0.135
		small	0.293	0.411	0.339
	HDLVS_sporadic	large	0.033	0.925	0.064
		small	0.080	0.309	0.126
PCA	1LV_all	large	1.000	0.618	0.762
		small	1.000	0.784	0.875
	3LVS_cluster	large	0.759	0.584	0.655
		small	0.705	0.707	0.703
	3LVS_over	large	0.866	0.440	0.581
		small	0.842	0.525	0.643
	HDLVS_interconnected	large	0.079	0.064	0.066
		small	0.420	0.306	0.349
	HDLVS_sporadic	large	0.036	0.073	0.045
		small	0.195	0.302	0.232

Table F: Evaluation metrics (srmr and nlog10(P)) summarised as mean over simulations for arbitrary confounding design with n=100.

**method**	**design**	**graph**	**srmr**	**nlog10P**
sim	DAG	large	0.077	10.126
		small	0.083	2.652
	HDLVS_interconnected	large	0.097	7.293
		small	0.163	1.993
	HDLVS_sporadic	large	0.092	9.264
		small	0.128	2.677
CGGM	DAG	large	0.077	10.153
		small	0.083	2.552
	HDLVS_interconnected	large	0.098	9.480
		small	0.119	3.075
	HDLVS_sporadic	large	0.101	11.702
		small	0.106	4.467
gLPCA	DAG	large	0.077	10.126
		small	0.083	2.656
	HDLVS_interconnected	large	0.079	6.930
		small	0.120	2.363
	HDLVS_sporadic	large	0.075	9.103
		small	0.099	3.207
LRpS	DAG	large	0.000	0.326
		small	0.064	0.224
	HDLVS_interconnected	large	0.000	0.427
		small	0.162	0.713
	HDLVS_sporadic	large	0.000	0.467
		small	0.128	0.348
PCA	DAG	large	0.051	10.836
		small	0.041	2.958
	HDLVS_interconnected	large	0.057	7.971
		small	0.077	2.571
	HDLVS_sporadic	large	0.056	9.757
		small	0.065	4.231
PCSS	DAG	large	0.018	2.616
		small	0.084	1.361
	HDLVS_interconnected	large	0.059	2.222
		small	0.193	2.748
	HDLVS_sporadic	large	0.052	2.589
		small	0.160	3.133
Trim	DAG	large	0.000	1.310
		small	0.056	1.646
	HDLVS_interconnected	large	0.000	1.656
		small	0.068	3.041
	HDLVS_sporadic	large	0.000	1.707
		small	0.046	2.768

Table G: Evaluation metrics (srmr and nlog10(P)) summarised as mean over simulations for dense confounding design with n=100.

method	design	graph	srmr	nlog10P
sim	1LV_all	large	0.326	4.249
		small	0.419	2.641
	3LVS_cluster	large	0.200	5.242
		small	0.222	2.898
	3LVS_over	large	0.211	3.713
		small	0.263	2.494
CGGM	1LV_all	large	0.044	4.162
		small	0.080	4.417
	3LVS_cluster	large	0.042	6.150
		small	0.100	5.420
	3LVS_over	large	0.046	4.871
		small	0.130	4.757
gLPCA	1LV_all	large	0.089	5.417
		small	0.068	3.436
	3LVS_cluster	large	0.121	6.691
		small	0.131	4.660
	3LVS_over	large	0.147	4.632
		small	0.168	3.266
LRpS	1LV_all	large	0.000	0.379
		small	0.074	0.369
	3LVS_cluster	large	0.000	0.348
		small	0.160	0.569
	3LVS_over	large	0.000	0.197
		small	0.152	0.442
PCA	1LV_all	large	0.045	2.587
		small	0.047	3.193
	3LVS_cluster	large	0.046	6.770
		small	0.048	4.961
	3LVS_over	large	0.046	4.709
		small	0.051	3.975
PCSS	1LV_all	large	0.052	7.735
		small	0.123	5.088
	3LVS_cluster	large	0.068	6.357
		small	0.179	5.237
	3LVS_over	large	0.090	6.135
		small	0.222	6.320
Trim	1LV_all	large	0.000	2.183
		small	0.025	3.968
	3LVS_cluster	large	0.000	2.255
		small	0.054	4.474
	3LVS_over	large	0.013	1.732
		small	0.099	4.324

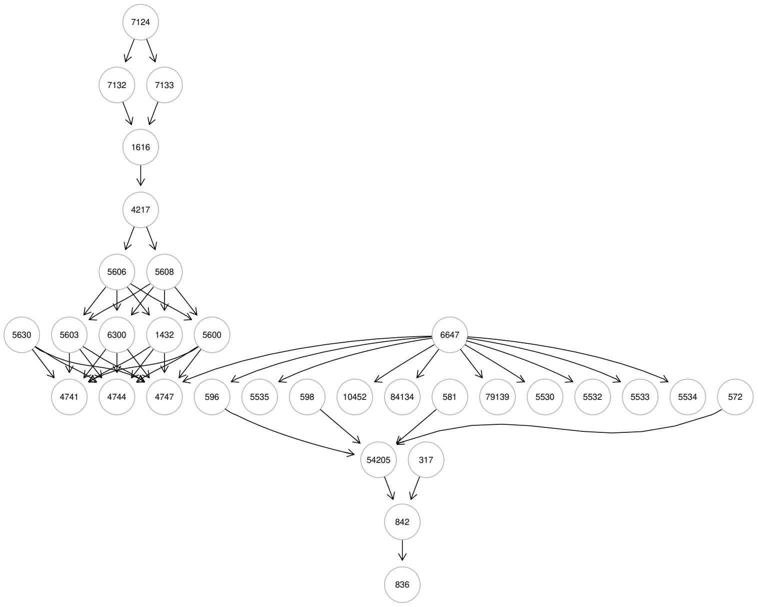

Fig A: Small subgraph from ALS pathway for simulated data (32 nodes and 47 edges).

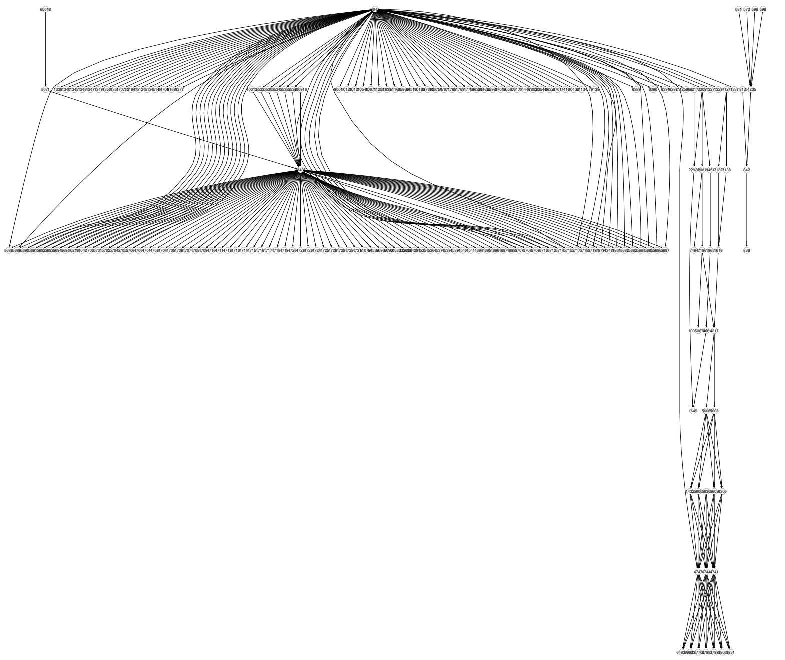

Fig B: Large subgraph from ALS pathway for simulated data (190 nodes and 259 edges).

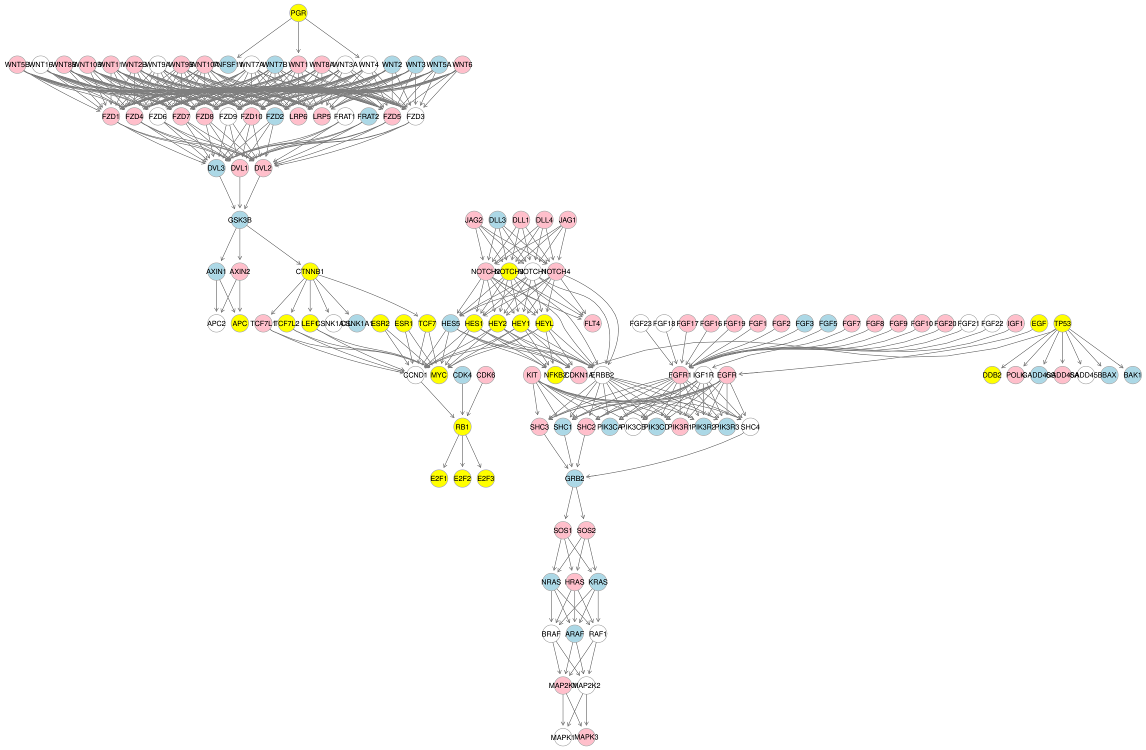

Fig C: Perturbed original subnetwork from benchmark data analysis. Nodes in the recovered subnetworks are coloured in yellow if they represent TFs, pink-shaded if significantly activated or blue-shaded if signficantly inhibited.

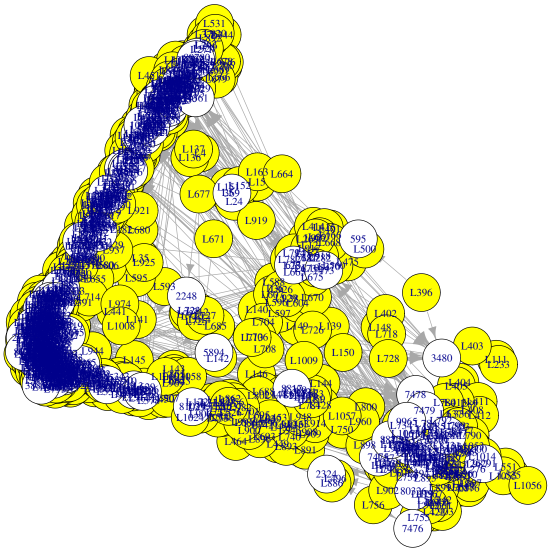

Fig D: Subnetwork of adjacency matrix obtained from d-separation tests of SEMbap() algorithm with yellow nodes corresponding to LVs in benchmark data analysis.

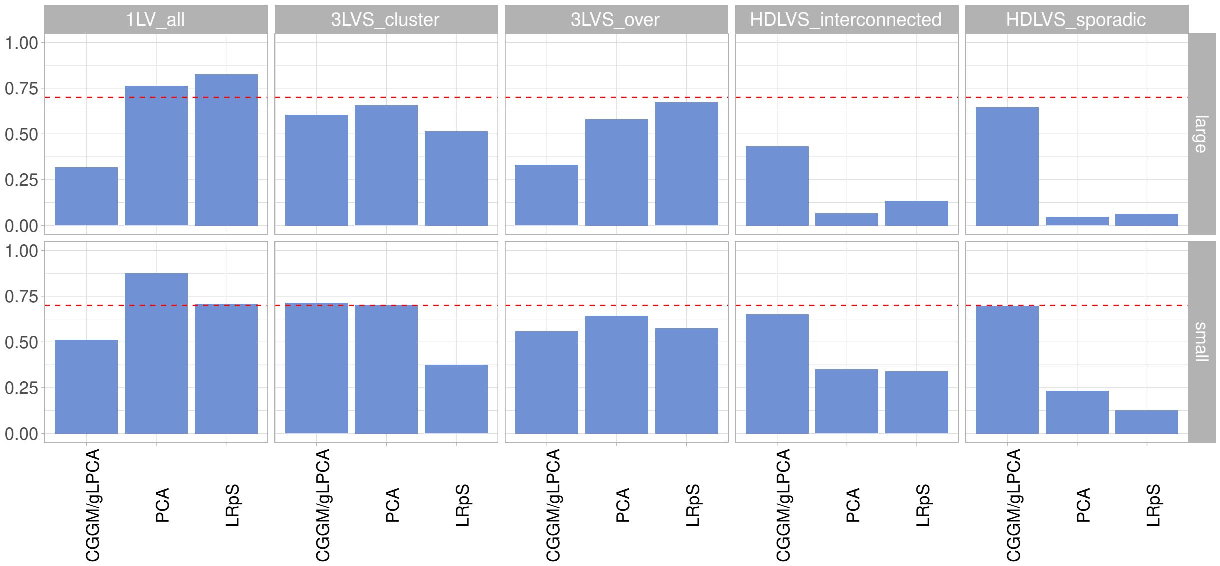

Fig E: F1-score summarised as mean over simulations for dense/sparse confounding design with n=100. For SVD methods with NULL confounding covariance, none performance metrics have been computed.

References

[1] Cevid D, Buhlmann P, Meinshausen N. Spectral Deconfounding via Perturbed Sparse Linear Models. Journal of Machine Learning Research. 2020;21(232):1–41.

[2] Agrawal R, Squires C, Prasad N, Uhler C. The DeCAMFounder: Non-Linear Causal Discovery in the Presence of Hidden Variables Journal of the Royal Statistical Society Series B: Statistical Methodology 2023;85(5):1639–1658

[3] Chandrasekaran V, Parrilo PA, Willsky AS. Latent variable graphical model selection via convex optimization. The Annals of Statistics. 2012;40(4):1935 – 1967.

[4] Frot B, Nandy P, Maathuis M. Robust causal structure learning with some hidden variables. Journal of the Royal Statistical Society: Series B (Statistical Methodology). 2019;81.
